# Variation in Organophosphate Pesticide Metabolites in Urine of Children Living in Agricultural Communities

**DOI:** 10.1289/ehp.6890

**Published:** 2005-01-10

**Authors:** William E. Lambert, Michael Lasarev, Juan Muniz, Jennifer Scherer, Joan Rothlein, Juanita Santana, Linda McCauley

**Affiliations:** ^1^Center for Research on Occupational and Environmental Toxicology, Oregon Health and Science University, Portland, Oregon, USA; ^2^School of Nursing, University of Pennsylvania, Philadelphia, Pennsylvania, USA; ^3^Oregon Child Development Coalition, Wilsonville, Oregon, USA

**Keywords:** agriculture, children, farmworkers, pesticides

## Abstract

Children of migrant farmworkers are at increased risk of exposure to organophosphate pesticides because of “carry-home” transport processes and residential location. Although this at-risk status is generally recognized, few available reports describe the extent of this exposure among agricultural communities. We quantified dialkyl phosphate (DAP) levels in serial samples of urine from 176 children, 2–6 years of age, in three Oregon communities hosting differing agricultural industries: pears, cherries, and fruit berries. Up to three spot samples of urine were collected from children at the beginning, mid-point, and end of their parents’ work seasons. The median levels of dimethylthiophosphate (DMTP), the most commonly detected metabolite, was significantly higher in urine samples from children in each of the three agricultural communities (17.5, 19.0, and 41.0 ng/mL) relative to a reference group of children who lived in an urban community and whose parents did not work in agriculture (6.5 ng/mL; Kruskal-Wallis, *p* < 0.001). After controlling for age, sex, and weight, the median level of DMTP in children in the pear community was 1.92 times higher than the level in children of the berry community [95% confidence interval (CI), 1.14–3.23] and 1.75 times higher than the level in children of the cherry community (95% CI, 0.95–3.23). We observed increasing levels of DMTP across the work season only within the berry community. Levels decreased in the cherry community and remained constant in the pear community. Substantial temporal variation within the children followed demonstrates the need for multiple urine samples to most accurately characterize longer term and/or cumulative exposure. The observed variability in urinary DAP levels, between communities and over time, could be attributed to the types and amounts of organophosphate pesticides used, the timing of applications and degradation of residues in the environment, work operations and hygiene practices, the proximity of housing to orchards and fields, or the movement of these working families. Additional studies of variation in pesticide exposure across agricultural regions are needed.

Measurement of dialkyl phosphate (DAP) compounds in urine has been used to assess exposure to organophosphate pesticides (OPs) in children living in rural agricultural settings (Azaroff 1999; Curl et al. 2002; Koch et al. 2002; Loewenherz et al. 1997; Lu et al. 2000; Shalat et al. 2003) and more recently in urban communities (Curl et al. 2003; Lu et al. 2001). These biomarkers provide an integrated estimate of exposure received through ingestion, inhalation, and dermal absorption during the 24–48 hr preceding testing (Feldman and Maibach 1974; Loewenherz et al. 1997). Because the analytical method used to quantify urinary DAPs is relatively new and technically difficult (Moate et al. 1999), data on the extent of OP exposure in various types of communities are limited.

Children have been the focus of many exposure assessments because their activity patterns, behavior, and diet lead to increased risk of exposure relative to adults (Eskenazi et al. 1999). The sensitivity of developing organ systems, specifically the brain and central nervous system, and immature detoxification and elimination capacities further increase children’s risk for adverse health effects (Faustman et al. 2000). In the United States, most children are probably exposed to OPs to some extent. Household surveys indicate extensive residential use of these compounds (Whitemore et al. 1994), and the potential for widespread low-level chronic exposure exists (Adgate et al. 2001). Children who live in agricultural communities are regarded to be at particularly high risk for exposure because of their proximity to fields and orchards where these chemicals are applied in high volume (Simcox et al. 1995). Additionally, children whose parents work in agriculture receive “carry-home” exposure via transport on their parents’ work clothing and shoes (Lu et al. 2000). Our studies in Oregon have characterized these exposure pathways in children of migrant farmworkers and have demonstrated elevated levels of residues in their residences (McCauley et al. 2001).

In this report, we describe the occurrence of DAP compounds in urine samples collected from children of migrant farmworkers in three separate communities that host differing agricultural industries using varying types of OPs, and a reference group of children living in a urban area.

## Materials and Methods

### Study design.

This study was conducted as a partnership between Oregon Health and Science University (OHSU) and the Oregon Child Development Coalition (OCDC), which is the grantee for Oregon Migrant Head Start. A cross-sectional design was employed to collect serial samples of urine from preschoolers attending Head Start programs at three centers operated by OCDC in the communities of Hood River, The Dalles, and Cornelius. For comparison purposes, a reference sample of preschool-age Hispanic children who lived in an urban area, Portland, and whose parents did not work in agriculture was also assembled. Urine was collected from the agricultural communities during June–September 2001, at the beginning, mid-point, and end of each work season. The timing of the sampling varied in each community depending on the time that the farmworkers began to arrive in the community, enrolled their children in Head Start programs, and started work harvesting crops. In the reference community group, samples of urine were collected during July and November 2001.

### Survey sites.

The communities selected for study are geographically separate and differ in the type of agricultural industry. Hood River primarily produces pears but also produces apples. Hood River is located along the Columbia Gorge, approximately 100 km east of Portland, Oregon. The farm workers in this community tend to be semipermanent residents who live in cabins, trailers, or apartments that are located in or alongside orchards. Azinphos-methyl [trade name Guthion; Chemical Abstract Service (CAS) No. 86-50-0], chlorpyrifos (CAS No. 2921-88-2), and phosmet (trade name Imidan; CAS No. 732-11-6) are used to control pests such as coddling moth and are applied May–August. Harvesting of tree fruit begins in August and extends through October. Urine samples were collected from the children at the Hood River Migrant Head Start day care centers in Parkdale and Odell in June, August, and October.

The second survey site was The Dalles, where cherries are grown. The Dalles is located along the Columbia Gorge, 30 km east of the Hood River community. Cherry harvest lasts only 1 month, usually beginning in mid-June. The cherries are hand harvested by migrant farmworkers, who live with their families in camps, cars, trailers, and tents. Chlorpyrifos can be applied to the cherry crop at prebloom in March by air blast method from the ground, whereas ultra-low-volume concentrate malathion (CAS No. 121-75-5) is applied aerially in late May and every 7–10 days throughout harvest. Urine samples were collected at the Dalles Migrant Head Start Center in June and July.

The third agricultural community, Cornelius, has both rural and suburban features and is situated in the northwest Willamette Valley. The farmworker population consists of settled farmworkers who work in the nursery, vineyard, and berry industries and a highly mobile migrant population of farmworkers who harvest the strawberry, caneberry, and blueberry crops during the summer months. The migrant population of farmworkers receive priority enrollment in Migrant Head Start and was targeted for this study. These families tend to live in camps composed of wood cabins and trailers that are adjacent to berry fields. Strawberry, caneberry, and blueberry growers apply diazinon (CAS No. 333-41-5), azinphos methyl, chlorpyrifos, and malathion to control pests. Urine samples were collected at the Cornelius Migrant Head Start Center the months of June, July, and August.

Reference subjects were recruited through Head Start and school-based programs in the Portland metropolitan area. Hispanic families were screened and invited to participate only if the parents reported that they did not work in agriculture, nursery, or landscaping businesses. Typical jobs held by families in the reference group include food service, factory work, and child care. These families lived in apartments in an urban setting.

### Recruitment and data collection.

Eligibility for participation was restricted to preschool-age children, 2–6 years of age, who were able to urinate while sitting on a toilet. Signed informed consent was obtained from parents as they registered their child for the Migrant Head Start Program. All participants except children from the reference population had at least one parent working in the field, orchard, or nursery while participating in the study. Demographic information was extracted from Head Start records, which were completed at time of registration. The extracted information included the child’s name, sex, age, date of birth, type of residence, previous and current place of employment, and crop worked by the parents. Reference subjects were selected from summer Head Start programs at three sites in northeast and north Portland, and Gresham (a suburban community east of Portland). When reference subjects were recruited and informed consent from the mother was obtained, each parent was asked questions to confirm that their child was between the ages of 2 and 6 years, and that neither the parents nor other adult household members worked in agriculture. At the time the urine sample was collected, participants were weighed.

### Human subjects review.

The study protocol and procedures for informed consent were reviewed and approved by the OHSU institutional review board (protocol 4216).

### Urine collection.

Urine samples were collected from each participant while the child attended Migrant Head Start. Research assistants collected the single void urine samples mid-morning through early afternoon using commode inserts. The urine was transferred into a urine specimen cup, labeled, and transferred on ice to the Center for Research on Occupational and Environmental Technology analytical laboratory.

### Urinalysis.

Urine specimens were adjusted to pH 3.0 and stored at −20°C until extraction and analysis. Five DAPs were analyzed by gas chromatography (GC): dimethylphosphate (DMP), diethylphosphate (DEP), dimethylthiophosphate (DMTP), diethylthiophosphate (DETP), and dimethyldithiophosphate (DMDTP). Urine samples were prepared for GC analysis according to a modified method of Moate et al. (1999). Aliquots of the samples underwent azeotropic distillation with methanol and evaporation under a nitrogen stream. Sample extracts were then derivatized with 2,3,4,5,6-pentafluorobenzyl-bromide to convert phosphate acids to esters. Extracted samples were analyzed on a gas chromatograph (model 5890; Hewlett-Packard, Palo Alto, CA) equipped with a pulsed flame photometric detector (OI Analytical, College Station, TX). The limit of detection (LOD) for each of the metabolites was calculated from the instrument response factor corresponding to a concentration having a peak area three times the baseline noise (blank signal). The specific LODs for the five metabolites were 4.0 ng/mL for DMP, 2.0 ng/mL for DEP, 2.2 ng/mL for DMTP, 1.6 ng/mL for DMDTP, and 1.6 ng/mL for DETP. The average extraction efficiencies of the five metabolites were, respectively, 87, 84, 97, 96, and 93%. Creatinine concentrations (micrograms per deciliter) were determined by the modified Jaffe rate method (Sigma Diagnostics Creatinine Kit no. 555; Sigma-Aldrich, St. Louis, MO).

### Quality control/quality assurance.

Quality control data generated for each set of urine samples were used to provide an overall assessment of precision, accuracy and overall reliability of the method. Spike sample recoveries and urine blank analysis were conducted for every set of 12 samples. Urine samples known to contain low levels of DAP were used for blanks and for spike recoveries. Urine samples were spiked with DAP reference standards varying in concentration from 2 to 50 ng/mL.

LOD is defined as the lowest concentration that can be determined to be statistically different from a blank. However, in practice, the detection of an analyte by an instrument is often based on the extent to which the analyte signal exceeds peak to peak noise (U.S. Environmental Protection Agency 2000). The LOD for each set of metabolites was calculated from the instrument response factor corresponding to a concentration having a peak area three times the baseline noise (signal) corresponding to the urine blank sample.

### Data analysis.

To obtain stable measurements of urinary metabolites, we averaged the concentrations measured in the second and third samples, corresponding to the mid-point and end of the work season. The first sample, collected at the time the child was being registered in Head Start, was not included because it could represent exposures that had occurred before the parents began working in agriculture in the area. The second sample was obtained at least 2 weeks into the work, reflecting exposures while the parents were working in the area, and the third sample, taken at the end of the work season, also reflected exposures occurring while their parents were working in the area.

We evaluated the distribution of creatinine levels and excluded urine samples less than the 5th percentile (14 mg/dL) and greater than the 95th percentile (110 mg/dL) from the analysis because of concerns of hydration state and metabolic disorders (Loewenherz et al. 1997; Lu et al. 2001). Tampering by dilution is unlikely given the young age of the subjects and the supervision of urine collection by our field staff.

During the course of the survey, urine samples were collected from a total of 214 subjects. Nineteen subjects were excluded from the statistical analysis because second or third samples were not obtained. Data for an additional 19 subjects were excluded because creatinine levels fell outside the range of 14–110 mg/dL. Statistical analyses were performed on a data set consisting of 176 subjects.

DMTP was the most commonly quantified DAP. The percentage of samples below the LOD was 20.5%. DMP and DMDTP were below the LOD in 33.0 and 58.8% of creatinine valid samples, respectively. For comparison, the ethyl compounds, DEP and DETP, were below LOD in 91.2 and 68.2% of samples, respectively. Because ethyl metabolites were detected with much less frequency, statistical analysis was restricted to the methyl metabolites. The infrequent detection of DEP and DETP in our survey is consistent with the experience of researchers at the University of Washington (Curl et al. 2002; Fenske et al. 2000; Lu et al. 2001). In our case, inclusion of the large proportion of unquantifiable observations would needlessly complicate analyses and not produce informative results.

Standardization of DAP metabolites to creatinine (nanomoles per gram creatinine) was performed in the statistical analysis. However, because this adjustment did not alter findings in any substantive way, we chose to present our findings in units unadjusted for creatinine (nanograms per milliliter and micromoles per liter).

We summed the molar equivalent concentrations of the methyl metabolites to create a combined methyl DAP measure. Nondetects were treated as zeroes in the summation of molar concentrations, and if the total methyl DAP value was zero, then it was assigned the value of 0.5 LOD for DMDTP (0.5 × 0.010 μmol/L), the methyl DAP with the lowest LOD.

### Statistical methods.

Demographic data were summarized using means ± SDs. Metabolite levels below the LOD were treated as 0.5 LOD for the purpose of statistical analysis and were summarized using the geometric mean, geometric SD, and percentiles. The geometric mean is a descriptive statistic suitable only for summarizing the data; however, it is an estimator for the population median. Hypothesis tests or confidence intervals based on the geometric mean make inference to the population median, and subsequent wording indicates the median is the parameter being tested/estimated. We used Kruskal-Wallis one-way rank analysis of variance to test for differences in the median concentrations of metabolites between the communities. We used separate Wilcoxon tests, with *p*-values adjusted by the method of Benjamini and Hochberg (1995), to determine which pairs of medians significantly differed at the 0.05 level. Within the three agricultural communities, potential confounding of the location effect by age, sex, and weight was controlled for using a general linear model (GLM) applied to the log-transformed data. After back-transformation, adjusted means from this model estimate the population median and additive changes become multiplicative effects (Aitkin et al. 1994; Ramsey and Schafer 2002). We used extra-sum-of-squares *F*-tests (Netter et al. 1989) to test significance of multiple effects within the GLM.

To analyze variation in metabolite levels within subjects and communities over time, DMTP concentrations were log-transformed, and paired *t*-tests were applied to assess differences between sample collection times: time 1 versus time 2 (beginning vs. middle of work season), time 2 versus time 3 (middle vs. end of work season), and time 1 versus time 3 (beginning vs. end of work season). We used Pitman’s test for correlated variances (Pitman 1939; Snedecor and Cochran 1980) to determine whether the amount of dispersion changed between two points in time. All analyses were performed using R version 1.9 (R Development Core Team 2004).

## Results

Table 1 presents the characteristics of the samples obtained in each community. The distribution of males and females did not vary across the four communities. Overall, 52.3% of the sample was female. There was indication that the weight of children differed significantly from one community to the next (*F*_3,210_ = 6.25, *p* < 0.01). Children from the pear community weighed significantly less than did children from either the cherry or reference community [95% confidence interval (CI), 3.6–10.4 lb less]. The mean age of children was not compared across communities because, by design, we sampled only children between 4 and 6 years of age.

In our comparisons of urinary metabolites, we averaged the concentrations measured in the second and third samples for each individual collected during the mid-point and end of their parents work season. The median concentrations of urinary DMTP and the combined methyl DAP by weight-volume concentrations units were significantly higher in the agricultural communities versus the urban reference community [Kruskal-Wallis test: χ*^2^* (3 df) = 27.29 and χ^2^ (3 df) = 25.237, respectively; *p* < 0.001 for both) (Table 2)]. The median concentrations of DMTP and combined methyl DAPs were significantly higher in children from the pear community, relative to those observed in either the cherry or the strawberry communities (adjusted *p*-value from Wilcoxon test = 0.008 for both). Median values in samples from the cherry and strawberry communities were not significantly different (adjusted *p*-value from Wilcoxon test = 0.786).

We used a GLM applied to log-transformed data to compare urinary DMTP and the combined methyl DAP levels across the agricultural communities. The analysis adjusted for age, sex, and weight. Means from this analysis (derived from β-coefficients) estimated the median effect in the population after they had been back-transformed. CIs for the effects were derived from CIs for specific β-coefficients and then back-transformed. The results indicated that the differences in the median level of DMTP in children of agricultural communities remained significant after controlling for age, sex, and weight (extra-sum-of-squares *F*-test, *F*_2,108_ = 8.270, *p* = 0.04). The median level in the pear community was 1.92 times higher than the level in the berry community (95% CI, 1.14–3.23). Median levels were 1.75 times higher in the pear community than in the cherry community (95% CI, 0.95–3.23), but this was only a suggestive difference (*p* = 0.075). The difference between the berry community and the cherry community was not significant when accounting for age, sex, and weight (*p* = 0.719). Controlling for age, sex, and weight reduced the statistical significance of the community effect for the combined methyl DAP data within the three agricultural communities (extra-sum-of-squares *F*-test, *F*_2,108_ = 2.165, *p* = 0.120).

Table 3 presents the geometric means and SDs for DMTP measurements by community and sample time. CIs for the effect were found by first calculating a confidence interval for the mean difference of the log-transformed data and then back-transforming the end points with corresponding inference made to median levels. In the berry community, concentrations tended to increase from beginning to end of the work season (Table 3). The median was estimated to be 2.18 times higher than the median at the beginning of the work season (95% CI, 1.26–4.08 times higher). In the cherry community, concentrations of DMTP sharply decreased from the beginning of the work season to the midpoint, 10 days later. At the end of the work season, three weeks after the initial sample, urinary DMTP began to increase. In the pear community, concentrations of DMTP did not change in an appreciable way from the beginning to end of the work season.

## Discussion

Our analysis of urinary biomarkers adds to a growing body of literature that suggests children of agricultural workers experience more exposure to OPs than do children who live in urban areas (Lu et al. 2000). The median level of DMTP, the most commonly detected metabolite, was significantly higher in the children of migrant farmworkers relative to urban Hispanic children whose parents did not work in agriculture. More important, however, is our demonstration of substantial variation in OP metabolite levels across communities hosting differing agricultural industries, and substantial variation within a community over time. This variation was demonstrated among communities within the same geographic region, and within relatively short work seasons lasting weeks to months, conditions that could be expected to foster homogeneity.

Median levels of DMTP differed significantly among communities, despite substantial interchild variability and overlapping distributions. Although the precise reasons for the observed differences between locations cannot be inferred from our data, we attribute the variation to differences in the types of pesticides used on the fruits and berries, and the timing of application and opportunity for environmental degradation before contact with workers at the time of harvest. For example, OPs are applied to pears as needed to control infestations until the time of harvest, whereas OPs are applied very early in the development of cherry fruit and not again, or infrequently, with months of time for breakdown of residues in sunlight and moisture. It is also possible that the greater extent of contact with foliage associated with picking pears (vs. cherries) may create greater opportunity for transfer of foliar residues to the clothing and skin of adults, who may carry these residues to their children. Finally, differences in type of housing and proximity of residence to orchards and fields may also explain the observed differences. The pear community is located in a valley, and air blast spraying and drift transport occurs near the homes of migrant farmworkers, possibly increasing the opportunity for exposure. Proximity of housing to application areas has been demonstrated to influence carpet residue levels in the homes of workers in Washington (Lu et al. 2000) and Oregon (McCauley et al. 2001). Furthermore, we have observed a larger number of detectable OPs in carpet dust of homes in the pear community (Hood River) compared with homes in the berry community (Cornelius, Washington County) (McCauley et al. 2001).

Koch et al. (2002) reported a temporal pattern of pesticide exposures in children living in an agricultural community over an entire year and the impact of agricultural spraying on exposure. The children studied in the Koch et al. (2002) report were enrolled via Women, Infants, and Children clinic populations in central Washington State. There are important differences in the design of these two studies. We were unable to study children over an entire year because, by the nature of their parent’s migratory work, they move on to other agricultural regions or return to their native country for a portion of the year. We were unable to assess the impact of spraying pesticides on urinary OP levels, because at the time the parents are harvesting the crops, the active spraying season is over. Instead, the goal of our study was to point to differences between the OP metabolite levels in children according to the type of harvesting work their parents were engaged in and to compare agricultural children to urban children.

The levels of urinary metabolites observed in the children of our pear community are similar to those reported for children of apple orchard workers living in central Washington State and extensively characterized by Fenske and colleagues (Loewenherz et al. 1997). The same OPs (e.g., azinphos methyl, phosmet) are applied to both crops to control coddling moth, using similar air blast spray systems, in similar settings of cultivation. In fact, apples are cultivated adjacent to pear orchards at our study site.

For reference, we collected urine samples from Hispanic children attending summer Head Start programs in the Portland, Oregon, metropolitan area. The geometric mean level of DMTP in this control group was 7.2 ng/mL. This level is slightly higher than the geometric mean of 2.7 ng/mL (95% CI, 1.85–4.01) reported for 471 children, 6–11 years of age, sampled in the 1999–2000 National Health and Nutrition Examination Survey [Centers for Disease Control and Prevention (CDC) 2003]. The difference may be attributed to age and differing hand-to-mouth behavior and floor contact in our younger children. Our combined methyl DAP median concentration was 0.15 μmol/L, very similar to the 0.11 μmol/L median reported for Seattle children 2–5 years of age (Lu et al. 2001). Presumably, the urinary metabolites observed in studies of urban children derive from dietary exposures to residues, and exposures associated with residential and public (schools, parks) applications.

Our serial sampling design provided an opportunity to consider temporal variation in urinary metabolite levels. Given the short half-life of 24–48 hr for DAPs, we expected to observe variation, specifically increased excretion of metabolites, as parents began work and started to transfer residues to the home environment. Further, we expected increasing concentrations as body burdens increased and new doses were superimposed on previous doses that were being metabolized and eliminated from the children’s bodies. We observed increasing levels of DMTP across the work season only in the berry community. Levels decreased at the cherry community and remained constant in the pear community. We attribute this pattern to differences in the migrant farmworker labor forces. In the berry community, migrant workers who arrived to work for the short harvest season apparently had low body burdens and low exposures to OPs in the period immediately before arriving. In the cherry community, most migrant farmworker families arrived directly from agricultural work in California and/or from the nearby pear community, and sufficient time had not yet passed for the OP metabolites to wash out of their bodies. In the pear community, the workers are settled and maintain their migrant status and Head Start benefits by returning to Mexico once per year in the winter. These workers and their children live for extended periods in the valley, in close proximity to the orchards and associated pesticides; therefore, their body burdens may reflect steady state, rather than new and accumulating doses. Although the differences that we observed could be attributed to differences in the work patterns and total exposure to OPs, it is possible that the different pharmacokinetics of specific pesticides used in these communities and their half-lives could have contributed to the observed difference.

Our findings are subject to several limitations. First, the DAP metabolites measured in this study represent a partial view of the total mix of pesticide exposures received by children. Exposures to other classes of pesticides and herbicides certainly occur, and the total exposure to all classes of agricultural chemicals is not quantified by our methods. Further, the sources and routes of exposure to OP compounds cannot be identified by measurement of urine DAPs, which provide an integrated indicator of exposure to a variety of OP compounds via ingestion, inhalation, and dermal exposure.

Despite these limitations, our survey is based on homogeneous samples of children who by virtue of their eligibility for enrollment in Migrant Head Start are of the same preschool age, share Hispanic ethnicity and come from the same socioeconomic class, and have at least one parent who works in agriculture. We collected measurements on age, sex, and weight and analyzed creatinine levels to control for physiologic variation. Appreciating the relatively short half-life of these metabolites, we collected serial samples from the children and used the average of two samples collected at the middle and end of the work season to investigate between community differences and to improve the characterization of longer-term exposure. We did not use the first urine sample, collected at the time of Migrant Head Start enrollment, because of concerns that this urine sample represented the exposure of the child before the family’s move to the new work location and Head Start center. Although some methyl DAPs probably come from exposure to OP residues on foods (Curl et al. 2003), this class of biomarker has proven to be a valid and reliable measure of exposure via other pathways, including hand-to-mouth transfer, dermal absorption, and inhalation, and the observed pattern of variation between communities is consistent with differing pesticide application practices by the agricultural industries in these areas. Although it was not practically possible to measure exposure to the full suite of chemicals to which these children are exposed, OPs as a class are among the most toxic chemicals in use by the agriculture industry and, as a class, present significant health risks.

In conclusion, our findings indicate that there is substantial variation in level of exposure to OPs among the children of migrant farmworkers living in different communities. This diversity in exposure experience must be considered in exposure assessments and health risk analyses. Failure to characterize potential differences between communities may introduce exposure misclassification into epidemiologic studies. Further, our observation of substantial temporal variation within a child supports the need for multiple urine samples to accurately characterize longer term and/or cumulative exposure.

## Correction

In the original manuscript published online, Michael Lasarev and William E. Lambert were listed as the first and second author, respectively. The order has been reversed here.

## Figures and Tables

**Table 1:** Age, sex, and weight of children from three agricultural communities and reference urban community in Oregon.

	Community				
Parameter	Reference (*n* = 65)	Berries (*n* = 63)	Cherries (*n* = 38)	Pears (*n* = 48)	Significance*^a^*
No. of samples/child					
Mean ± SD	1.2 ± 0.4	2.9 ± 0.4	2.8 ± 0.5	2.6 ± 0.7	—
Min, max	1, 2	1, 3	1, 3	1, 3	
Age [years (mean ± SD)]	NA*^b^*	4.2 ± 1.0	4.3 ± 1.1	4.2 ± 1.0	—
Sex (% male)	51	44	50	46	χ^2^(3) = 0.66 *p* = 0.88
Weight [lb (mean ± SD)]	42.1 ± 9.6	39.2 ± 10.2	42.6 ± 11.2	35.9 ± 7.5	*F*_3,210_ = 6.25 *p* < 0.01

Abbreviations: max, maximum; min, minimum; NA, not applicable.

a Number of samples per child and age were controlled by design and not tested.

b Date of birth not collected.

**Table 2:** Comparison of child urinary DMTP (ng/mL) and combined methyl*^a^* (μmol/L) DAP metabolites*^b^* among three agricultural communities and referent community.

	Reference (*n* = 61)		Berries (*n* = 52)		Cherries (*n* = 29)		Pears (*n* = 33)	
Summary statistics	DMTP	Combined methyl*^b^*	DMTP	Combined methyl	DMTP	Combined methyl	DMTP	Combined methyl
Geometric mean*^c^*	7.25	0.12	18.81	0.25	20.24	0.26	38.54	0.40
Geometric SD*^d^*	5.33	3.80	3.43	2.54	2.51	1.93	3.07	2.34
10th percentile	1.10	0.01	4.48	0.09	5.60	0.12	7.68	0.11
25th percentile	1.10	0.06	7.97	0.12	9.40	0.15	27.00	0.27
50th percentile	6.50	0.15	17.50	0.23	19.00	0.25	41.00	0.44
75th percentile	30.00	0.30	40.50	0.46	37.00	0.39	87.00	0.77
90th percentile	61.00	0.50	99.90	0.90	63.00	0.53	126.00	1.00

a “Combined methyl” is the summed molar equivalent concentration of DMP, DMTP, and DMDTP (μmol/L).

b Concentrations are the average of the mid- and the end-of-season samples (ng/mL).

c Back-transformed mean of log-transformed data (an estimate of the median).

d Back-transformed SD of log-transformed data.

**Table 3:** The amount and variation of DMTP levels from children in agricultural communities across three time points in a harvest season.

	Geometric mean (geometric SD)			*p*-Values for comparison of DMTP levels*^a^*			*p*-Values for comparison of variation*^b^*		
Community	T1	T2	T3	T1 vs. T2	T1 vs. T3	T2 vs. T3	T1 vs. T2	T1 vs. T3	T2 vs. T3
Berries (*n* = 50)	7.2 (5.0)	9.6 (5.5)	15.7 (4.3)	0.34	0.02	0.10	0.67	0.54	0.30
Cherries (*n* = 29)	43.4 (2.6)	14.0 (2.5)	18.3 (4.0)	< 0.01	0.01	0.33	0.90	0.06	0.04
Pears (*n* = 31)	22.4 (3.4)	22.5 (5.3)	22.8 (5.4)	0.99	0.95	0.97	0.10	0.08	0.95

Abbreviations: T1, time 1; T2, time 2; T3, time 3. The amount and variation are summarized in terms of the geometric mean and geometric SD; *p*-values show whether the changes are significant.

a Based on paired *t*-test of log-transformed data.

b Based on Pitman’s test for correlated variances (performed on log-transformed data).
